# Recruitment of the cardiac conduction system for optimal resynchronization therapy in failing heart

**DOI:** 10.3389/fphys.2022.1045740

**Published:** 2022-12-15

**Authors:** Zhongli Chen, Xiaohong Zhou, Xuan Ma, Keping Chen

**Affiliations:** ^1^ State Key Laboratory of Cardiovascular Disease, Arrhythmia Center, Fuwai Hospital, National Center for Cardiovascular Diseases, Chinese Academy of Medical Sciences and Peking Union Medical College, Beijing, China; ^2^ Medtronic, Inc, Dublin, Ireland; ^3^ Department of Magnetic Resonance Imaging, Fuwai Hospital, National Center for Cardiovascular Diseases, Chinese Academy of Medical Sciences and Peking Union Medical College, Beijing, China

**Keywords:** heart failure, cardiac resynchronization therapy, biventricular pacing, His bundle pacing, left bundle branch area pacing

## Abstract

Heart failure (HF) is a leading health burden around the world. Although pharmacological development has dramatically advanced medication therapy in the field, hemodynamic disorders or mechanical desynchrony deteriorated by intra or interventricular conduction abnormalities remains a critical target beyond the scope of pharmacotherapy. In the past 2 decades, nonpharmacologic treatment for heart failure, such as cardiac resynchronization therapy (CRT) *via* biventricular pacing (BVP), has been playing an important role in improving the prognosis of heart failure. However, the response rate of BVP-CRT is variable, leaving one-third of patients not benefiting from the therapy as expected. Considering the non-physiological activation pattern of BVP-CRT, more efforts have been made to optimize resynchronization. The most extensively investigated approach is by stimulating the native conduction system, e.g., His-Purkinje conduction system pacing (CSP), including His bundle pacing (HBP) and left bundle branch area pacing (LBBAP). These emerging CRT approaches provide an alternative to traditional BVP-CRT, with multiple proof-of-concept studies indicating the safety and efficacy of its utilization in dyssynchronous heart failure. In this review, we summarize the mechanisms of dyssynchronous HF mediated by conduction disturbance, the rationale and acute effect of CSP for CRT, the recent advancement in clinical research, and possible future directions of CSP.

## Graphical Abstract

**Figure:**
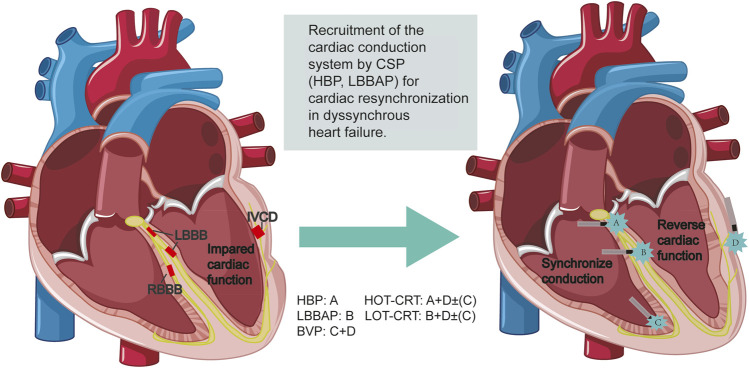
Emerging strategies for cardiac resynchronization for dyssynchronous heart failure.

## Introduction

Heart failure (HF) is a global health burden with increasing morbidity and mortality with a 1-year mortality rate of 10–35%. (Ambrosy et al., 2014). Even with guideline-directed medical treatment (GDMT) (Huang H. T. et al., 2022), a significant proportion of patients remain symptomatic with irreversible reduced left ventricular ejection fraction (LVEF). While multiple underlying causes (volume overload, inflammation, ischemia, neuroendocrine disorders) contribute to chronic heart failure, ventricular dyssynchrony, caused by impaired cardiac conduction system, is another underlying mechanism occuring in 24%–47% of the heart failure patients with reduced ejection fraction (HFrEF) patients and is often refractory to pharmacological therapies. (Lund et al., 2013; Prinzen et al., 2022). The so-called ventricular dyssynchrony refers to the discoordination of the electrical activation and mechanical contraction within or between the ventricles. Impaired cardiac conduction system disturbance, including left bundle branch block (LBBB), right bundle branch block (RBBB), and intraventricular conduction delay (IVCD), presents with wide QRS complex and is associated with the development of heart failure.

To correct electrical dyssynchrony in heart failure patients with wide QRS complex and reduced LVEF, cardiac resynchronization therapy (CRT) *via* biventricular pacing (BVP) was introduced in early 2000 and has brought remarkable benefits to HF prognosis, including a reduction of the all-cause mortality by 29% (Rivero-Ayerza et al., 2006) and heart failure hospitalization (HFH) rate from 0.338 to 0.204 events per patient-year (Varma et al., 2021). However, despite tremendous efforts, the traditional BVP approach of CRT is also facing challenges including difficulties in left ventricular (LV) lead positioning and a non-responding rate of approximately 30% (Kaza et al., 2022). Therefore, efforts have been made to pursue optimal electrical and mechanical synchrony through the conduction system pacing (CSP) *via* direct activation of the His bundle or left bundle branch, which can restore the functionality of impaired cardiac conduction system so as to produce physiological ventricular activation propagation and better mechanical synchrony. More recently, evidence for clinical utility of CSP for CRT accumulates, providing prospect of CSP in positive modulation of the failing heart. Herein, we discuss the ventricular conduction disturbance-mediated dyssynchrony in the deterioration of HF, the rationale of CSP for CRT, the recent clinical evidence for potential indications for patient selection and future directions of CSP.

## Progress in assessment of ventricular dyssynchrony

Ventricular dyssynchrony can be recognized through different assessments. Both electrical and mechanical synchrony can be measured directly through ventricular endocardial mapping and catheterization. But these measurements are invasive, risky, operator-dependent, and time-consuming, which limits their use in routine clinical practice. Therefore, non-invasive methods have been the mainstream for synchrony evaluation. The most commonly used non-invasive tool to quantify electrical synchrony is the 12-lead electrocardiogram (ECG.) The QRS duration (QRSd), left ventricular activation time in lead V5 or V6 and the QRS morphology have been adopted for a long time to assess the electrical dyssynchrony prior to or after the CRT. Another simple parameter is the ECG-derived QRS area, which provided a strong association with CRT response (Emerek et al., 2019). Recently, ECG imaging (ECGi), ECG belt, and ultra-high-frequency ECG (UHF-ECG) have been used as non-invasive tools that provide more detailed information about ventricular activation. Not only the right ventricle (RV) and LV dyssynchrony parameters can be assessed separately (for example, the standard deviation of activation times, left ventricular dyssynchrony index (LVDI), LV/RV total activation/depolarization time), but also the interventricular dyssynchrony can be evaluated (eg: e-DYS) (Mizner, 2022).

Additionally, mechanical dyssynchrony can be measured using Doppler echocardiography, 2-dimensional (D) specking-tracking echocardiography, 3D echocardiography, or the cardiac magnetic resonance (CMR) strain analysis. Several qualitative markers such as septal flash and pre-systole rebound stretch, and quantitative indices like the peaking time, excursion amount, or myocardial wasted work can be calculated (Fang et al., 2010; Zweerink et al., 2018). The commonly used LVEF, LVESV, beat-to-beat blood pressure, (Arnold et al., 2018), and rate of LV pressure rise (expressed as dp/dt max) (Kato et al., 2022)do not directly reveal the mechanical dyssynchrony, but represent the structural or functional status associated with the electrical and mechanical dysynchrony, thus they are usually applied in the clinical evaluation of CRT benefits.

## The pathophysiological mechanism of dyssynchronous heart failure

### LBBB morphology and cardiac dyssynchrony

Asynchronous ventricular activation and contraction are associated with cardiac dysfunction. Clinically, the most prominent form of the underlying conduction disturbance is LBBB, followed by non-specific IVCD and RBBB. Previous studies have demonstrated the causal relationship between conduction disturbance and cardiac remodeling, especially LBBB in regulating electromechanical dysynchrony and impairing cardiac function (Vernooy et al., 2005; Byrne et al., 2007; Vernooy et al., 2007). Specifically, in LBBB, the rapid intrinsic conduction in LV is impaired, and ventricular activation starts from the right ventricle, then to the LV endocardium, in which electrical activation propagates *via* the working myocardium. It has been reported that in true or complete LBBB, trans-septal conduction time takes 30–40 m and the LV free wall is activated even later, resulting in the marked prolongation of LV activation time (LVAT) and a wide-notched QRS complex (Auricchio et al., 2004), followed by mechanical dyssynchrony (Kroon et al., 2015).

**FIGURE 1:**
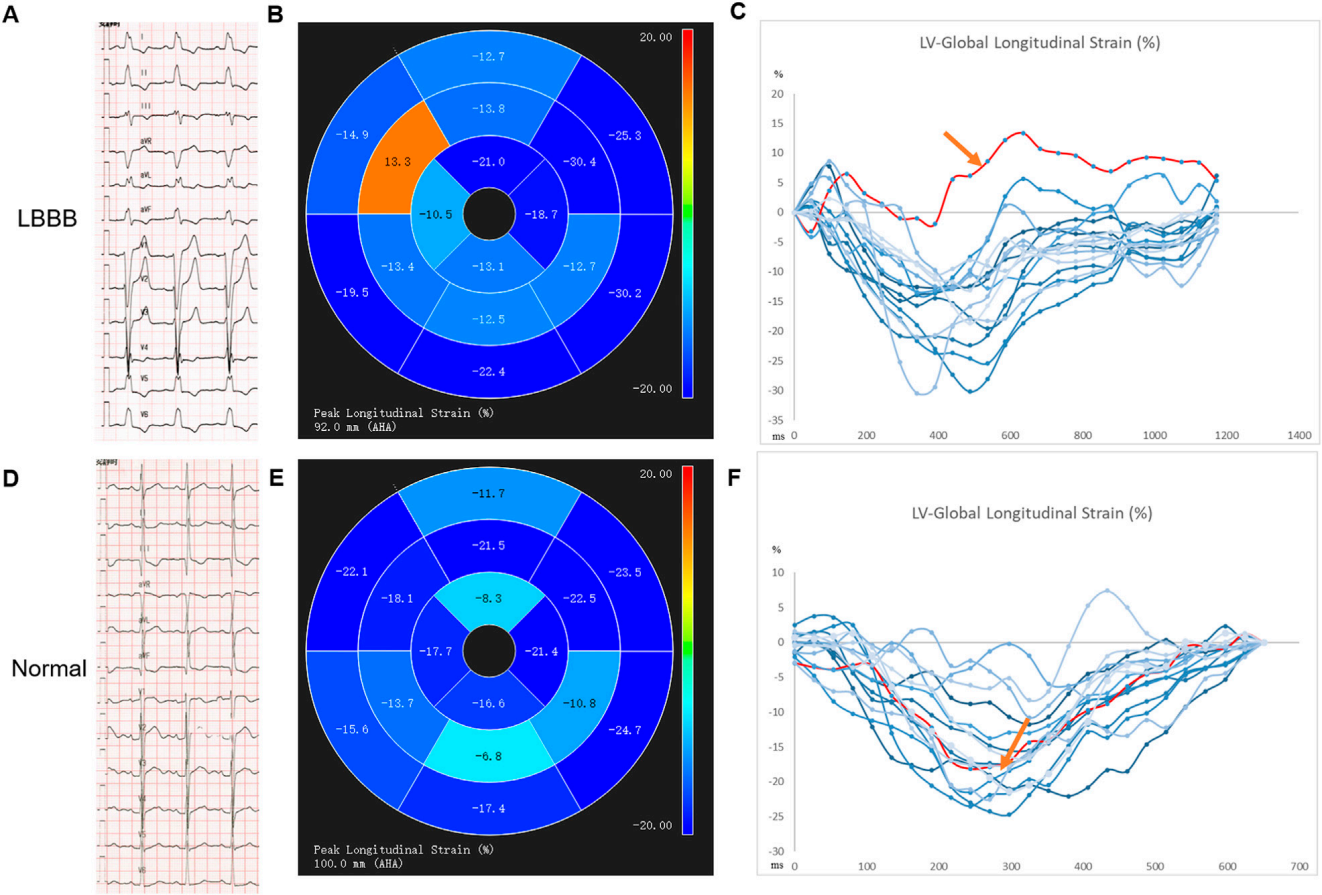
The impaired global longitudinal strain of the septal segment of a LBBB patient with preserved LVEF by CMR-feature tracking technique. **(A–C)** an example from an LBBB patient: A electrocardiogram of the LBBB; B-C AHA segment diagram of 2D long axis GLS, segment eight in septum shows abnormal GLS with a positive value, represented the contradictory motion of septum, red spline; an example from a healthy control patient by **(D–F)**: D electrocardiogram of normal intrinsic rhythm, **(E–F)**: AHA segment diagram of 2D long axis GLS, the same segment in septum shows normal GLS with a negative value, represented as red spline.

For example, echocardiographic studies indicate that in LBBB, early activation of the right ventricle free wall (RVFW) and later contraction of LVFW causes the septal flash and pre-systole septal systolic rebound stretch, leading to supranormal contraction of the latest activated LVFW (Walmsley et al., 2016). Similar motion abnormalities of the septum were also evaluated by CMR (Figure 1). The myocardial work redistributes, so does the blood flow, and more wasted work is done by the ventricular myocardium, making energy metabolism more inefficient (Russell et al., 2012). As a consequence, LV structure and function are impaired as displayed by the rightward shift of the pressure-volume (PV) loop, larger LV end-diastolic volume (LVEDV), lower strains, and reduced LVEF. (Vernooy et al., 2005; Smiseth and Aalen, 2019).

### Non-LBBB morphology and dyssynchrony

The non-LBBB conduction disturbances such as RBBB and IVCD, which together account for about one-third of patients with QRS complex widening, represent the severity of the myocardial disease, inducing cardiac asynchrony and deteriorating cardiac function. RBBB, featuring evident delayed activation of RVFW segment (Dou et al., 2009), is related to a predominant reduction of RVEF and all-cause mortality in patients with and without heart failure (Gaba et al., 2020; Lai et al., 2020). Human studies in patients with RBBB and HF found that total/regional LV endocardial activation time of RBBB patients did not differ significantly from that in LBBB patients (Fantoni et al., 2005), and a higher prevalence (50%) of mechanical dyssynchrony was detected compared with the non-RBBB control group (Sillanmäki et al., 2020). But the specific mechanism of the RBBB-induced dyssynchrony remains to be elucidated.

IVCD, which always accompanies LBBB, holds heterogeneous delayed activation in various ventricular myocardial segments (Viswanathan et al., 2006). Different from LBBB, IVCD is characterized by multiple LV breakthroughs along the septum with the presence of heterogeneous localized areas of late activation along the LV free wall (Derval et al., 2017). Therefore, though a less remarkable degree of RV/LV dyssynchrony has been observed in IVCD compared with BBB, significant intraventricular dyssynchrony exists (Kerwin et al., 2000). IVCD reflects the diseased structural substrate of the myocardium and serves as a predictor of a higher risk of both cardiovascular death or HF hospitalization and all-cause mortality in HFrEF patients (Nguyên et al., 2018; Kristensen et al., 2020).

### Pacing mediated dyssynchrony in patients with synchronous heart failure

RV pacing mimics the LBBB-type activation pattern even in patients with narrower intrinsic QRS complex. However, the LV mechanical dyssynchrony pattern in RV pacing is not identical to intrinsic LBBB. Specifically, in patients with RV-pacing-induced LBBB, the mid- and apical septal regions are activated earliest while in patients with intrinsic LBBB, the basal septum is activated earlier. Furthermore, an even higher degree of mechanical delay was reported in the lateral segments among the RV-pacing-induced LBBB pattern compared to the intrinsic LBBB (Ghani et al., 2011). Therefore, long-term RV pacing can decrease effective myocardial work and depress pump function (Tanaka et al., 2010; Ghani et al., 2011; Naqvi and Chao, 2021).

## The rationale, electrical and hemodynamic effects for conduction system pacing in CRT

The traditional BVP significantly normalizes the total activation time, inter-ventricular electrical coupling, and mechanical synchrony in patients with LBBB (Kerwin et al., 2000; Ploux et al., 2015) and hence provides clear clinical benefits in many clinical trials. However, BVP still brings non-physiological ventricular activation patterns due to pacing at two separate non-physiological sites, e.g., one in the LV epicardial site and the other in RV endocardial site. The study by Nguyên UC, et al. found that on the LV surface, there were curvilinear activation delays near the LV pacing site, forming the island of early activation. (Nguyên et al., 2018). Despite the reduction of LV activation delay in the LBBB group, the RV total activation time also increased (Nguyên et al., 2018). The randomized clinical trial also demonstrated that BVP could have detrimental effects in heart failure patients with narrow QRSd (Moss et al., 2009). In patients with narrow QRS complex or IVCD, a significant increase of total LVAT was reported, further proving the dyssynchronous electrical ventricular activation brought by BVP, as compared with the normal intrinsic conduction, which at least partly explains the detrimental effect of BVP-CRT on the hemodynamic response in patients with little or no electrical dyssynchrony (Ploux et al., 2015).

### Effect of HBP on electrical and mechanical synchrony

In comparison to BVP, CSP directly stimulates the native specialized conduction system, allowing for complete restoration of electrical depolarization and repolarization and leading to true physiological resynchronization. The rationale of His bundle pacing (HBP) for correcting LBBB mainly originates from the longitudinal dissociation hypothesis described by Narula in 1977, who revealed that before the separation of bundle branches, individual bundle branches also existed in a single cable within the His bundle (Narula, 1977). Therefore, LBBB with lesions within the His bundle can be corrected by pacing the distal region of the block area of the His bundle (HB). In 2005, the first case report of HBP in a 62-year-old female patient with reduced left ventricular ejection fraction (LVEF: 35%), LBBB [QRS duration (QRSd):160 ms], and left ventricle asynchrony was initiated by Dr. Vázquez (Morina-Vazquez et al., 2005). They got a constant capture of HB at an output of 1.6 V at 0.5 ms with an accompanying significant reduction in QRSd (30 m shorter than intrinsic QRSd). After a 6-month follow-up, the HBP threshold was stable at 2 V at 0.5 m and echocardiographic findings demonstrated a minimal delay of the left lateral wall, indicating that HBP could correct the electrical and mechanical dyssynchrony in HF. Later, studies detailing the electrical activation and the acute hemodynamic patterns of CSP and comparative analyses of CSP and BVP were performed. HBP, as described by electrophysiological studies in both animals and humans, provides a short total activation time (TAT), narrow QRS complex, and activation sequence most similar to normal physiological sinus activation. A more physiological ventricular activation pattern of HBP over BVP was also reported in LBBB patients using non-invasive epicardial mapping *via* 252-electrode ECGi vest and computer simulations (Arnold et al., 2018; Strocchi et al., 2020) The study reveals that HBP delivers a greater reduction of QRS duration, shorter LVAT, and better LV synchrony (evaluated by significantly reduced LVDI). The shortening of ventricular activation in HBP is also associated with incremental acute hemodynamic response, as supported by increased systolic blood pressure in HBP compared with the LBBB and the BVP groups (Arnold et al., 2018).

Additionally, HBP contains two subtypes of capture, one is by exclusive stimulation of the intrinsic His bundle, which is called selective HBP (S-HBP); the other is through activation of both the His bundle and the local myocardium, known as the non-selective HBP (NS-HBP)]. Whether one is superior to the other in terms of electrical and mechanical synchrony raises discussion. In a non-invasive epicardial electrical mapping study of 20 patients (60% LBBB, 10%RBBB), it is found that S-HBP and NS-HBP displayed similar LV activation patterns, whereas NS-HBP displays early activation in the basal to the mid-region of RV due to the capture of local para-Hisian myocardium. However, LVAT is preserved and RVAT is not significantly prolonged in NS-HBP compared with S-HBP, implying that a minor difference in electrical depolarization may not pose a great impact on the overall activation of ventricles in either S-HBP or NS-HBP (Arnold et al., 2021). Hemodynamic improvements are also found similar in both S-HBP and NS-HBP in patients with the narrow QRS complex. The echocardiographic measurements reveal that compared with RV pacing, both S-HBP and NS-HBP result in better inter and intra-ventricular synchrony without differences between the two groups (Catanzariti et al., 2006). But the non-inferior effect of NS-HBP to S-HBP can be explained by the capture of the conduction system because even pacing in the same para-Hisian area, pure myocardial pacing without the capture of intrinsic His-Purkinje system still leads to substantial QRSd prolongation and interventricular dssynchrony (Zhang et al., 2018; Curila et al., 2020). Therefore, it is reasonable to consider that although there are some differences in early activation sites, NS-HBP may not result in great electrical dyssynchrony or clinically different hemodynamic improvements when the conduction system is stably captured.

### Effect of LBBAP in electrical and mechanical synchrony

LBBAP, compromising the left ventricular septal myocardial pacing (LVSP) and direct capture of the left bundle branch (LBBP), offers another choice of CSP with a relatively lower and stable capture threshold and capacity of bypassing the pathological lesion and capturing the nearby conduction branch to overcome Infra-Hisian “distal” LBBB that cannot be corrected by HBP with a low capture threshold. (Upadhyay et al., 2019a).

**FIGURE 2:**
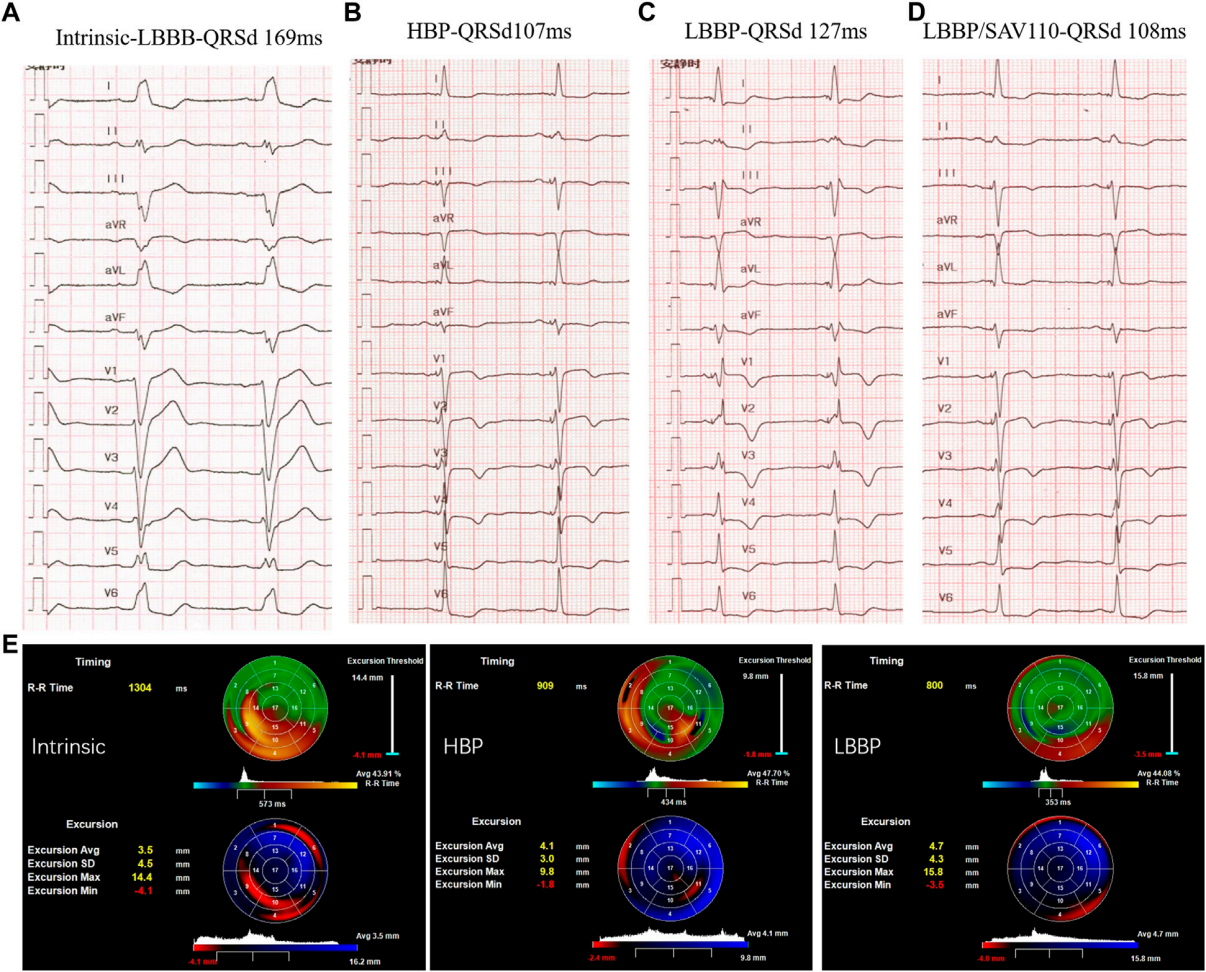
Comparison of LBBP and HBP in electrical and mechanical synchrony in a HF patient with LBBB: **(A)** Intrinsic rhythm with QRS duration (QRSd) of 169 ms; **(B)** Selective HBP at left bundle branch block (LBBB) correction threshold of 3 Vat 0.5 ms with QRSd equal to 107 ms. **(C)** LBBP at 0.5 V at 0.4 ms with QRSd of 127 ms and RV conduction delay pattern. **(D)** LBBP in DDD mode with SAV delay 110 ms with QRSd reduced to 108 ms. **(E)** 3-dimensional echocardiogram between selective His bundle pacing (HBP) and left bundle branch pacing (LBBP). Right: intrinsic rhythm; Middle: HBP in VVI mode; Left: LBBP in VVI mode. The upper “bull’s eye” depicts the timing of contraction green areas represent synchronous areas, blue areas contract early and red/yellow areas show late contraction. The lower “bull’s eye” shows the wall excursion with dark/red areas referring to reduced excursion and bright blue areas indicating the largest systolic radial excursion.

Recent preclinical and clinical investigations have delineated the effects of each subtype of LBBAP on electrical and mechanical synchrony and further compared these characteristics with both HBP and BVP. One computer simulation study indicates that LBBP and HBP are superior to BVP-CRT with a greater reduction of LVAT. However, interventricular synchrony of LBBAP is not as ideal as HBP due to a longer RVAT, but this can be mitigated by optimizing AV delay or bilateral bundle area pacing (Lin et al., 2020; Strocchi et al., 2020). The same electrical effect is also observed in our initial experience with an HF patient with LBBB who underwent successful implantation of both HBP and LBBP leads (Figure 2). With optimization of the sensed AV delay, LBBP produced equally narrow QRSd as HBP, with a stable lower LBBB correction threshold at implantation and after a 3-month follow-up. An echocardiographic analysis also suggested that either LBBP or HBP significantly alleviated the delayed activation and increased the average LV excursion.

Subsequent clinical studies further confirm the beneficial hemodynamic effect of LBBAP compared with HBP or BVP in both AVB and LBBB patients. Hu and others reported similar improvement of mechanical dispersion in both LBBP and HBP groups after 3-month follow-up while LBBP had better pacing parameters and shorter procedure time (Hu et al., 2021). By used two-dimensional speckle tracking echocardiographic imaging, they found LBBAP delivered a greater reduction of QRSd, reduced global wasted work, improved cardiac work efficiency, and led to better mechanical synchronization and efficiency than the traditional BVP-CRT (Liu et al., 2021).

Notably, the electrical and mechanical superiority among LVSP, S-LBBP and NS-LBBP have also been heatedly discussed. In a study comparing ventricular depolarization of LBBAP and HBP using UHF-ECG in bradycardia patients, both the NS-LBBP and LVSP led to longer septal and RV depolarization duration compared with HBP. Compared with NS-LBBP that preserves physiological LV depolarization but increases interventricular electrical dyssynchrony, the LVSP tended to preserve the interventricular synchrony while prolonged the depolarization time of the LV lateral wall (Curila et al., 2021a). Another study delineated that LBBP offered more significant ventricular synchrony with significantly decreased QRS area which was almost equal to normal ventricular activation when compared to LVSP and RV pacing, whereas the LVAT and QRS vector did not differ between the LVSP and LBBP and normal ventricular activation. Non-etheless, a recent study used UHF-ECG to analyze the superiority of LVSP over LBBP reported that LVSP from the proximity to the LBB region preserved interventricular dyssynchrony (described by e-DYS, the difference between the first and last activation in UHF-ECG), which was better than both S-LBBP and NS-LBBP (e-DYS: LVSP vs. NS-LBBP, vs. NS-LBBP: 16 m vs. -24 m vs-31 m), but did not prolong LV lateral wall activation (described by width of the UHF-QRS complex at 50% of its amplitude, Vd) among bradycardia patients. The study also compared S-LBBP and NS-LBBP in electrical activation and synchrony and found that S-LBBP produced shorter QRSd but led to greater interventricular dyssynchrony than NS-LBBP (Curila et al., 2021b).

Taken together, HBP and LBBAP are compelling alternatives for CRT with more physiological electrical and better hemodynamical effects than BVP. Though LBBAP does not capture the right side of the conduction system, it guarantees almost equal physiological LV activation as compared with HBP and can produce a similar narrow QRS complex and mechanical synchrony by optimizing the AV delay. In both types of CSP, recruitment of at least part of the conduction system enables shorter LV activation duration and narrower QRS duration. LVSP seems to induce better interventricular electrical synchrony compared with LBBP, but whether such minor differences in electrical synchrony can translate into clinical differences remains to be evaluated.

## Conduction system pacing: The evidence for clinical efficacy

### Application of HBP for patients with intrinsic conduction disturbance and indication for traditional CRT

Early in 2013, Barba-Pichardo pioneered a prospective study for HBP in 16 patients with LBBB and successfully achieved permanent HBP in nine patients, with mean QRSd shortening from 166 ms to 97 ms. After a mean follow-up time of 31.3 months, they reported a significant improvement in clinical and remodeling parameters of LV function (Barba-Pichardo et al., 2013). The first large sample size multi-center study was reported by Sharma et al. They assessed the feasibility and efficacy of HBP as a rescue strategy or a primary alternative to BVP in a group of systolic heart failure patients with LVEF lower than 50%, among whom 45% held BBB and 39% had RV pacing. After a mean follow-up of 14 months, both the rescue and the primary HBP groups showed a significantly narrowed QRSd and increased LVEF in both the LBBB (26%–41%) and non-LBBB morphology (32%–49%) groups (Sharma et al., 2018a). This research group further proved the efficacy of HBP for improving electrical synchrony and LV function in 39 patients with RBBB and reduced LVEF. They reported a significant reduction of QRSd from 158 to 127 m and observed an increase of LVEF from 31% to 39% after a mean follow-up of 15 months (Sharma et al., 2018b). These results provide a cornerstone for future randomized controlled trials in evaluating HBP as an alternative to BVP in patients who failed LV pacing and as a primary option for CRT. Regarding the long-term effect, Huang and others published 3-year results of a single-center prospective study of HBP for CRT in heart failure patients with typical LBBB and demonstrated the stable LBBB correction threshold along with the improvement in reverse remodeling echocardiographic metrics and clinical response (Huang et al., 2019). Subsequently, numerous observational studies of HBP for CRT were published, most of which corroborated these findings of the effective electrical synchrony, and functional and clinical improvement especially in patients with LBBB.

### HBP for patients with pacing-induced dyssynchrony CRT upgradation

For those with chronic RV pacing or intranodal block, early observational studies also proved HBP as an applicable approach for normalizing QRS complex and T waves. Furthermore, in those with pacing-induced cardiomyopathy (PICM) and decreasing LVEF, HBP was also feasible with a highly successful implantation rate of nearly 90% and induced a significant improvement of LVEF, NYHA class, while alleviating mitral valve regurgitation and brain natriuretic peptide levels (Shan et al., 2018; Vijayaraman et al., 2019a). The latest report compared the efficacy of HBP and BVP in patients with PICM and found HBP brought more considerable improvement in LVEF and more significant reverse remodeling than BVP, indicating the potential of HBP as an alternative to BVP for CRT upgrading among PICM patients. (Gardas et al., 2022).

### Clinical comparison of HBP and BVP

**FIGURE 3:**
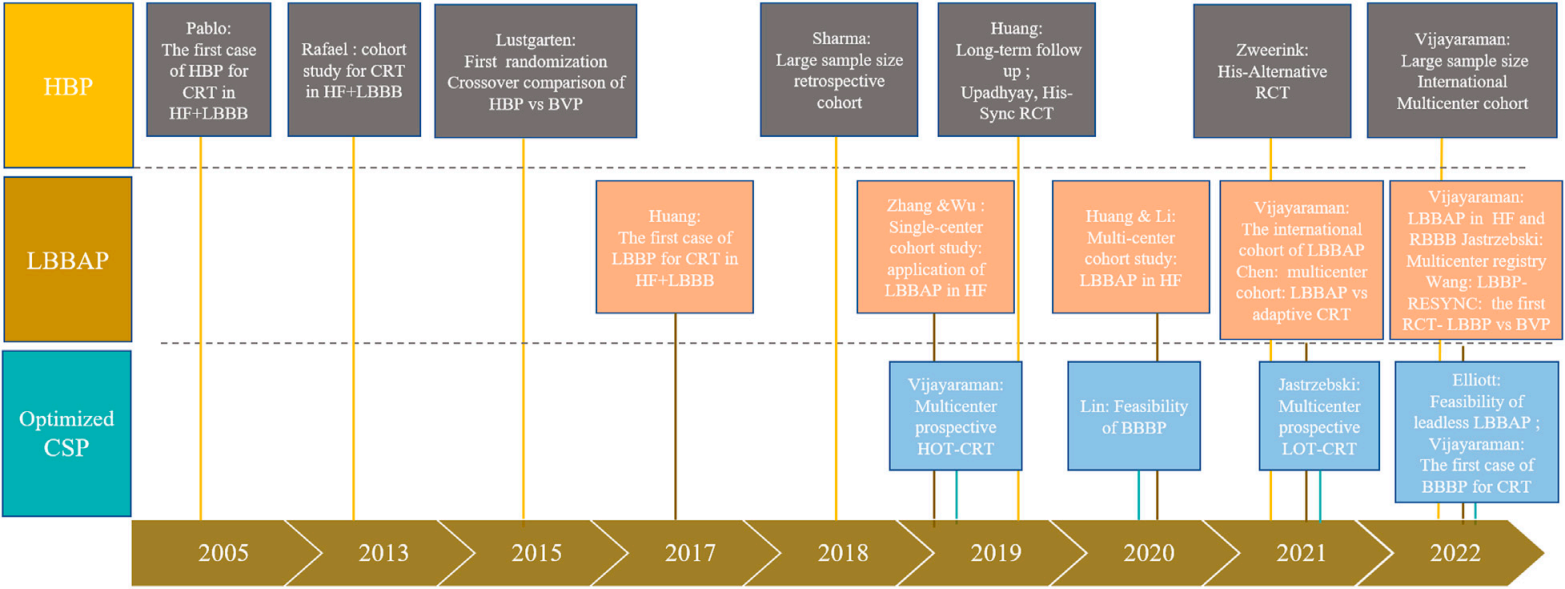
Landmark studies of CSP for CRT.

Compared with BVP, HBP appears to display more benefits in terms of acute hemodynamic improvements and echocardiographic response according to early results of small observational studies. The first randomization cross-over investigation comparing HBP and BVP for CRT was reported by Lustgartenl in 2015 (Lustgarten et al., 2015), in which in 12 patients who completed the entire protocol, the LVEF, NYHA, and quality of life scores all improved from baseline but did not differ between the HBP and BVP groups. The subsequent His-Sync study is the first multi-center randomized controlled trial (RCT) comparing HBP *in lieu* of BVP (Upadhyay et al., 2019b; Upadhyay et al., 2019c). The study randomized 41 patients with CRT indication to HBP-CRT or BVP CRT group. In the intention-to-treat (ITT, sample size in HBP vs. BVP: 21 vs. 20), treatment received (TR, sample size in HBP vs. BVP: 16 vs. 24), and per-protocol (PP, sample size in HBP vs. BVP: 11 vs. 14) analyses, HBP resulted in narrower QRSd compared with the BVP group but the LVEF improvement and echocardiographic response rate did not show a significant difference despite the numerically higher improvement of LVEF and a trend towards higher response rate in HBP group in PP analysis. However, numerous shortcomings of the study were identified including a small sample size, high bi-directional crossover rate, and broad criteria for patient selection (especially IVCD), limiting the power and evidence sufficiency of the results. The His-alternative CRT is another randomized study comparing HBP and BVP for symptomatic HF patients with Strauss LBBB. 50 patients were randomized to the HBP and BVP group with a cross-over rate of 28% from HBP to BVP. ITT analysis demonstrated non-superior effect of HBP to BVP in QRSd narrowing or echocardiographic response, while PP analysis showed higher LVEF and lower LVESV after 6 months of follow-up in the HBP group than in the BVP group. Although the evidence generated to date is insufficient to claim that HBP is superior over BVP for CRT, these results provide the potential for better electrical synchrony of HBP and laid a foundation for HBP as an alternative to BVP (Supplementary Table S1, Figure 3).

### Application of LBBAP for patients with intrinsic conduction disturbance and indication for traditional CRT

Given the drawbacks of a higher pacing capture threshold and a relatively long learning curve in HBP, LBBAP, with a lower capture threshold and higher success rate, sheds light on CSP for CRT. The landmark study of LBBAP for CRT was conducted by Dr. Huang in 2017 in a heart failure (LVEF of 32%) patient with typical LBBB (QRSd 180 ms). After the failure of left ventricular lead placement, HBP also failed to correct the LBBB at an output of 10 V. LBB was captured at 0.5 V at 0.5 ms, after optimization of atrioventricular (AV) delay, QRSd was reduced to 94 ms and the threshold was stable after a 1-year follow-up, with significant improvement of LVEF (32%–62%) and NYHA class (IV to I) (Huang et al., 2017). Since the anatomic merits of LBB fan-shape distribution (Ponnusamy et al., 2020), LBBAP has been widely applied in different centers rapidly while clinical evidence has been accumulated since 2017 (Supplementary Table S2, Figure 3). In 2019, two single-center observational studies carried out respectively by Zhang and Wu documented a significant reduction in QRSd and improvement in cardiac function after a mean follow-up of 6.7 and 32.5 months (Wu et al., 2019; Zhang et al., 2019), providing promising evidence for LBBAP feasibility and efficacy as a CRT approach in HF patients with LBBB. Afterward, a multi-center prospective cohort study by Huang and Li further confirmed the effectiveness of LBBAP in improving electrical synchrony and functional improvement of heart failure with LBBB (Huang et al., 2020; Li et al., 2020). A larger international multicenter cohort study initiated by Vijayaraman and coworkers analyzed the LBBAP in a board population with LVEF <50% and indication for CRT (including both LBBB and non-LBBB patients). They found in both the LBBB and non-LBBB group, the LBBAP provided significant QRS narrowing (LBBB 162 ms–133 ms; non-LBBB 160 ms to 143 ms, *p* < 0.01) and improved clinical and echocardiographic outcomes (NYHA class: LBBB 2.8 to 1.7, non-LBBB 2.7 to 1.8; LVEF: LBBB 30%–44%; non-LBBB 33%–43%, all *p* < 0.01).

The latest clinical evidence also suggested LBBAP in the population of HF patients with RBBB. Though the QRSd only showed a modest reduction, LBBAP was still associated with improvement in LVEF and NYHA class, indicating that LBBAP might be a choice of alternative CRT for patients with cardiac dysfunction and RBBB (Vijayaraman et al., 2022).

### LBBAP for patients with pacing-induced dyssynchrony and CRT upgradation

Similar to HBP, LBBAP was also proved feasible for patients with pacing-induced cardiomyopathy (PICM) and RV pacing upgrading in small sample studies (Li et al., 2021; Qian et al., 2021; Chen et al., 2022). Permanent LBBAP was successfully achieved in 93%–100% of patients with PICM and could also be performed safely in those with intranodal blocks. After the follow-up time ranging from 6 to 12 months (Qian et al., 2021; Rademakers et al., 2022), LBBAP could result in significant narrowing of QRSd, and improvement of LVEF and NYHA function with no observations of upgrade-related complications. But clinical observations comparing the efficacy of BVP and LBBAP for CRT upgradation are still lacking.

### Clinical comparison among LBBAP, HBP, and BVP

The first multi-center comparison of LBBAP and optimized BVP was reported recently by Chen et al. (Chen et al., 2022) to compare LBBAP with BVP with the adaptive algorithm in HF patients with LVEF ≤35% and LBBB. The results revealed a better electrical and mechanical resynchronization and higher super-response rate of LBBAP compared to BVP (Chen et al., 2022).

In a non-randomized treatment investigation comparing treatment outcomes of LBBAP, HBP, and BVP among patients with HFrEF and typical LBBB (Wu et al., 2021), similar improvements in symptoms and LV function were observed between LBBAP and HBP groups that were better than BVP. A recently published large multicenter cohort study in a large sample size of HF patients with LVEF lower than 35%, and CRT indications (in which 87 underwent HBP, 171 underwent LBBAP, 258 underwent BVP) found significantly narrower QRS complex, greater improvement of LVEF and lower rates of death or HFH during a mean follow-up of 27 months in patients receiving CSP as compared with BVP. But no significant differences in death or HFH were observed between the HBP vs. LBBAP group. In the latest study including patients with AF after atrioventricular junction ablation, LBBP held higher successful implantation rates, better pacing parameters, and fewer lead-related complications compared with HBP, though both achieved similar improvement in clinical outcomes (Cai et al., 2022). More recently, the first RCT (LBBP-RESYNC trail) to compare CRT efficacy between LBBP and BVP among heart failure patients with non-ischemic cardiomyopathy and LBBB found more improvement in LVEF by LBBP-CRT than BVP-CRT after 6-month follow-up (Wang et al., 2022).

As for the different clinical effects between LBBP and LVSP, there is still no head-to-head comparison. The study by Jiang et al. found that both the LBBP and LVSP groups significantly lowered the incidence of heart failure hospitalizations and all-cause mortality in LBBB patients with baseline LVEF higher than 35% compared with patients with LVEF lower than 35% during 12 months-follow-up (Jiang et al., 2022). Another study including patients with LBBB also reported improvement of cardiac functional parameters in LBBAP (LBBP and LVSP) groups in patients with LVEF lower than 50% after a 6-month follow-up (Shan et al., 2021). But these studies did not specify the improvement in each group for comparison. Only in the sub-group analysis of one published study, those who underwent LBB optimized CRT (LOT-CRT) with LBB capture showed better echocardiographic (11.1% vs. 4.7% of LVEF improvement, *p* = 0.0196) and clinical response (82% vs. 61%, *p* = 0.035) than the LVSP group, indicating that capture of LBB might provide a clinical benefit over LVSP (Jastrzębski et al., 2021). Recently, Jimenez et al. reported a significant improvement in LVEF and a decrease in LVESV following LBBP but diminished LVEF and increased LVESV in those without LBB capture in a small group of patients with a comparable baseline LVEF and wide QRSd (Ramos Jimenez et al., 2022). So far, the evidence is still lacking, and comparable studies or randomized trials are warranted for comparing the long-term clinical effects between LBBP and LVSP.

### Optimal lead position

The quest for the optimal lead position is based on clinical evaluations of CSP as a novel approach to CRT. The optimal lead position should preserve or restore the functionality (ventricular electrical synchrony) of the cardiac conduction system with consideration of technical efficiency and pacing parameters. CSP introduces better LV electrical synchrony with a narrow QRS complex compared with traditional BVP, with HBP displaying more physiological activation similar to the normal intrinsic activation in the absence of relative RV delay observed in LBBAP. Regarding the implantation process and pacing parameters, LBBAP can be a technically more promising way due to the shorter learning curve, higher successful implantation rate and stable pacing parameters when compared to HBP. But recently reported distal HBP may overcome these drawbacks through deep septal His-bundle capture (Supplementary Table S3) (Vijayaraman, 2020).

Besides, the evaluation of the optimal lead position may be individualized and tailored for a different population. Current clinical evidence from small RCTs and observational studies suggest that LBBAP may bring a higher improvement of LVEF, and a similar survival rate compared with BVP in patients with NICM and LBBB as compared with patients with non-LBBB and/or ICM. However, clinical evidence of the efficacy of CSP compared with BVP among patients with non-LBBB morphology is limited. Therefore, CSP may be best suited for LBBB patients while the BVP might be more appropriate for those with non-LBBB pattern, but more clinical evidence in patients with non-LBBB morphology is required (Wouters et al., 2021; Strocchi et al., 2022).

Previous studies and guidelines suggest that in patients with reduced LVEF and narrow QRS complex, BVP provides limited benefit (Moss et al., 2009; Tracy et al., 2012). Compelling results of applying CSP in patients with PICM, RV pacing upgrading as well as AV node ablation in atrial fibrillation patients are accumulating (Vijayaraman et al., 2017; Cai et al., 2022; Huang W. et al., 2022; Ivanovski et al., 2022). Hence, we expect that CSP may be a better option for primary and upgrading therapy in HF patients who have intact intraventricular conduction but need high RV pacing burden due to bradycardia or AV node ablation.

## Optimization of CSP

### His-optimized CRT

The prerequisite of CSP is to place the pacing lead tip at the appropriate site of the conduction system. However, the coexisting IVCD can delay the activation of the myocardium segments, hampering the full correction of electrical disturbance. Hence, the His and LBB-optimized CRT have been introduced to further narrow the QRS complex by stimulating both the native conduction system and the later activated myocardial areas. Vijayaraman performed the His-optimized CRT (HOT-CRT) in 27 patients with LVEF≤35% and LBBB/IVCD that could not be fully corrected by HBP alone and observed a remarkable reduction in QRSd from baseline 183 m–120 m by HOT-CRT (HBP plus LV pacing) than the BVP (mean QRSd 162 m) or HBP alone (mean QRSd 151 m). They also found that HOT-CRT brought significant clinical and echocardiographic response rates of 84% and 92% respectively (Vijayaraman et al., 2019b). By using ECGi, Alwin Zweerink further found that the HOT-CRT appeared to bring more synchronous activation, as compared with BVP-CRT (including multipoint pacing, MMP) and HBP-CRT, and not only remarkably increased the ventricular electrical synchrony by reducing LVAT (LVAT reduction: HOT vs. HBP: 17 m, HOT vs. BVP: 22 m, HOT vs. MMP: 11 m) but also improved RV synchrony in RBBB patients (Zweerink et al., 2021).

### LBB-optimized CRT

The feasibility of LBB-optimized CRT (LOT-CRT) was conducted by a multicenter observational study in 112 patients with CRT indication, including 42% LBBB, 22% IVCD, 23% RV pacing, and 12% RBBB. LOT-CRT resulted in acutely improved electrical resynchronization, with the reduction of QRSd three times greater than BVP pacing and superior LVAT compared with LBBAP alone (mean QRSd: baseline 182 m, BVP 170 m, LBBAP 162 m, LOT-CRT 144 m) as well as improvement of LVEF (from 28.5% to 37.2%) and NYHA class (from 2.9 to 1.9) (Jastrzębski et al., 2021).

**FIGURE 4:**
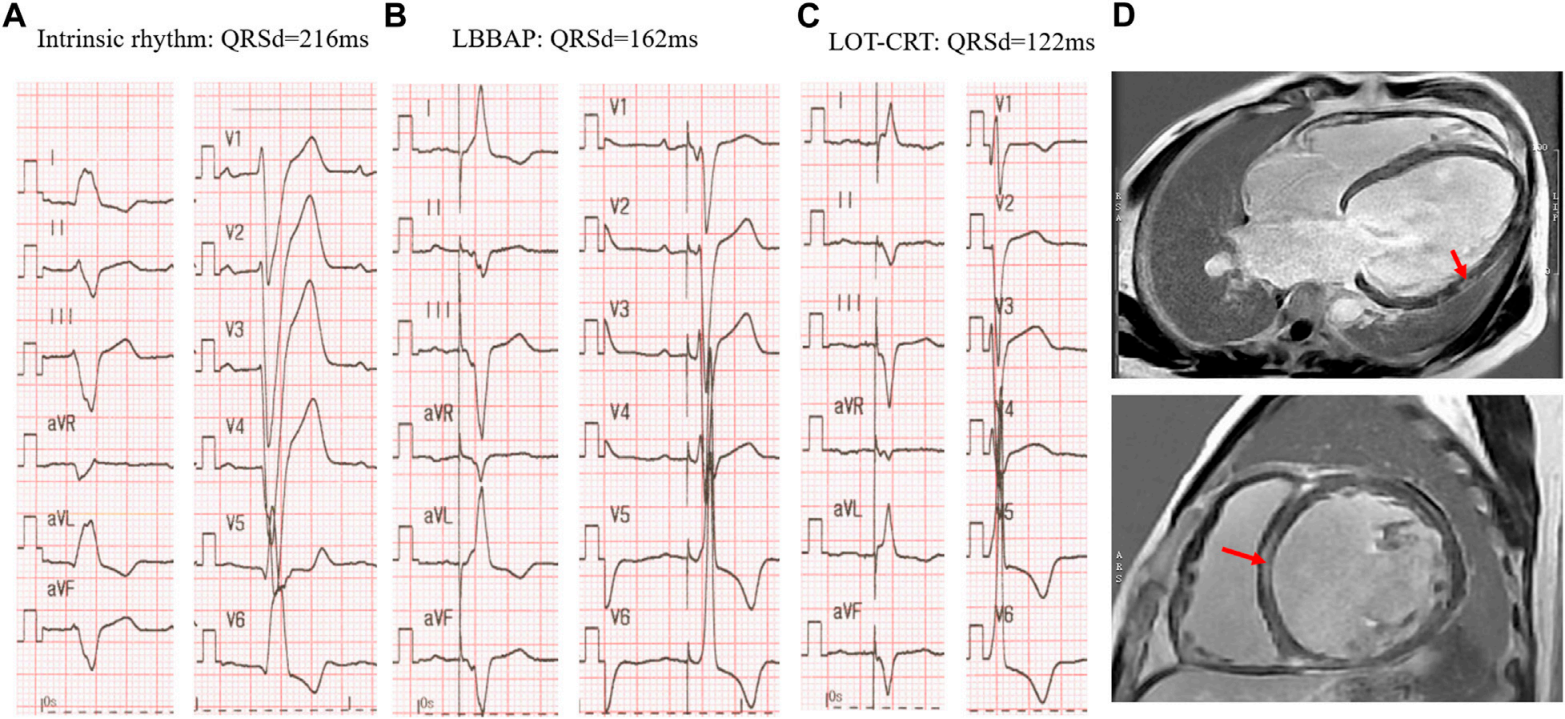
LOT-CRT for further correction of LBBB in a patient with myocardium scars. **(A)** Intrinsic rhythm with LBBB morphology and very wide QRS complex of 216 ms; **(B)** LBBAP alone at 3v at 0.4 ms in DDD mode with SAV of 120 ms; **(C)** LOT-CRT at 3.5 Vat 0.4 ms in DDD mode with SAV of 120 m and LBBAP prior to LV pacing of 60 ms. **(D)** Myocardial scars detected by CMR-late gadolinium enhancement prior to operation: upper LV four-chamber view, the arrow represents the scar in LV lateral wall; lower, short axis view, arrow indicates the septal scar.

These findings suggest that in patients with more advanced dysfunction of the conduction system/heart muscle as evidenced by wider baseline QRSd (eg.>180 m) or myocardial scars (Figure 4), despite proximal HB or LBB capture, additional LV pacing may be required to further correct the ventricular delay and achieve better cardiac synchrony as well as functional improvement.

### Bilateral bundle branch area pacing

**FIGURE 5:**
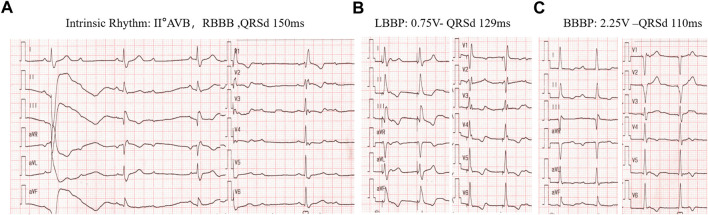
BBBP for correction of RBBD induced by LBBAP in a patient with intrinsic RBBB rhythm. **(A)** intrinsic rhythm of RBBB pattern with II° AV block and QRSd of 150 ms. **(B)** left bundle branch pacing by pacing the cathodal tip electrode alone at 0.75v at 0.4 ms with the RV delay pattern. **(C)**, Bilateral bundle branch area pacing (BBBP) with the cathodal tip electrode and the anodal ring electrode at 2.25 v at0.4 ms with QRSd further reduced to 110 ms.

As described above, although LBBAP normalizes LVAT, it also creates significant right ventricular conduction delays compared with normal intrinsic rhythm or HBP. Therefore, effects have been made to diminish the RBBB during LBBP to obtain better interventricular synchrony. Despite the previously discussed optimization of AV delay for fusing intrinsic RV conduction with LBBP, in 2020, a new concept of bilateral bundle branch area pacing (BBBP) was initiated by Lin et al., which involves simultaneous stimulation of both the left branch bundle area and the right branch bundle area. With BBBP, the RBBB pattern brought by LBBP was resolved and delayed right ventricular activation was diminished with significantly shorter QRSd as compared with LBBP. (Lin et al., 2020). Such a strategy may be particularly important for optimizing the electrical synchrony in those with intrinsic RBBB (Figure 5), among whom the existing RV delay may not be diminished by programming AV delay alone.

More recently, Vijayaraman delineated another way of BBBP by the direct right bundle pacing and left bundle pacing *via* two leads with a lower capture threshold (1-2v) in a 78-year-old HF patient with LBBB, and achieved complete right and left ventricular electrical resynchronization with a QRS complex similar to that of HBP at high threshold (8 V) (Vijayaraman, 2022).

Nevertheless, bilateral bundle branch area pacing still needs further investigation. The anatomical characteristics of the RBB, which, unlike the left bundle branch, is a cord-like thin structure with a shorter intramuscular course within the septum and then distributes in sub-endocardium region of the RV (Padala et al., 2022). Therefore, compared with the fan-shaped LBB, the pacing of the RBB is more difficult. It has been reported that the transition of QRSd during distal RBBP can be more pronounced in the frontal QRS axis, thus being more likely to be missed during threshold testing (Burri and Zimmermann, 2021). However, whether pacing the RBB area can bring more benefit to the electrical and mechanical synchrony, especially in those who have intrinsic RV electrical or mechanical delay still needs to be evaluated.

### Leadless LBBAP

Another compelling innovation, leadless LBBAP, which combines the attractive concepts of CSP and leadless pacing was initiated by Elliott, et al., in 2021. They provided the technical feasibility of leadless LBBAP to achieve better electrical synchrony from the LV septal pacing with the WISE-CRT delivery system (Elliott et al., 2021). A multi-center study further provided the feasibility and efficacy of leadless LBBAP *via* the WISE-CRT delivery system in two swine models and eight HF patients with wide QRSd. Preclinical data suggest the possibility of electrode tines in pacing the LV septal close to Purkinje tissue. All patients had the LV septal electrode and WISE-CRT implanted successfully, and temporary LV pacing significantly reduced the QRSd from 187.1 m to 139.8 m. At an early follow-up of 82.5 days, the median LV pacing percentage was 98.5%, and 62.5% of patients had symptom improvement (Elliott et al., 2022). The board distribution of the LBB conduction network provides histological merits for leadless LBBAP. It may be a promising option for patients with venous approach issues. Future studies are required regarding the long-term safety and efficacy of the technique, stable capture of the conduction system, and resynchronization effect compared with BVP-CRT or lead-based CSP.

The above studies or cases in optimization and innovation of CSP are conceptually attractive, but long-term clinical consequences or accumulative experiences for safety and efficacy remain to be validated in the future.

## Consideration of device programming

Since there are no CRT devices particularly designed for CSP, the programming of CSP remains confusing and challenging. Experience derived from previous clinical practice may be considered when programming biventricular devices.

Specifically, in patients with sinus rhythm and an atrial lead, the HBP or LBBAP lead is often connected to the LV port with RV backup pacing lead or defibrillation lead connected to the RV port, which can be used for ventricular sensing. Sequential pacing can be programmed with the CSP as a priority. Considering that anodal capture can attenuate the delay of RV activation in some patients, the bipolar configuration can be programmed for LBBAP (Lin et al., 2020). Moreover, adequate AV delay programming after the procedure allows the fusion of LBBAP with native right bundle conduction to provide another option to minimize the delay of RV activation (Raymond-Paquin et al., 2021).

For patients with atrial fibrillation, the CSP lead can be connected to the atrial port, with the RV and LV leads being connected to the corresponding RV and LV ports for HOT-CRT or LOT-CRT (Vijayaraman et al., 2019b; Zweerink et al., 2021). For HOT-CRT, the empiric value of HBP-VP delay of 60 m was reported to generate better HBP and ventricular pacing (LV, RV, BVP) fusion with shorter LVAT (Zweerink et al., 2021). More recently, the combination of adaptive CRT algorithm with LOT-CRT was proved feasible in patients with reduced LVEF and LBBB, which was associated with shorter paced QRSd, LVAT, and significant improvements in clinical NYHA and LVEF compared with BVP-CRT (Feng et al., 2022).

Nevertheless, there is still no clinical evidence to verify which pattern of device programming is optimal for CSP. Besides the successful implantation, clinical pitfalls should be evaluated and automatic device settings designed for CSP are urgently needed to ensure efficient CSP for CRT.

## Current recommendation and future directions

Providing the attractive concept of physiological pacing and initial encouraging results from multiple clinical observations, CSP has updated the conception of CRT for the treatment of electrical dyssynchrony-caused HF. Currently, guidelines from the American Heart Association (AHA) and the European Society of Cardiology (ESC) had also emphasized its role as a promising approach for CRT. In the 2018 AHA guidelines, HBP is recommended as a Class II indication for patients with AV block and LVEF between 36% and 50%, with an expected RV pacing rate over 40% (Kusumoto et al., 2019). In the 2021 ESC guidelines, HBP is recommended as a Class II recommendation as a bail-out for CRT candidates with unsuccessful coronary sinus lead placement (Authors/Task Force Members et al., 2022). By contrast, the newly released Chinese expert consensus on His-Purkinje conduction system pacing takes more proactive attitude towards the usage of both forms of CSP, revealing that CSP may be considered as a rescue approach for traditional CRT-non responders or a primary approach for CRT among HF patients with LBBB, QRSd over 130 m, LVEF lower than 35% and NYHA class II-IV after GDMT (Chinese Society of Pacing and Electrophysiology and Chinese Society of Arrhythmias, 2021).

However, whether CSP can serve as a primary CRT as BVP-CRT in routine clinical practice for patients with CRT indications needs more clinical evidence. Furthermore, for CRT response whether the previously established metrics of BVP can be used with HBP or LBBAP is unknown. The similarity and differences in characteristics of the target population appropriate for BVP and CSP remain to be addressed. Finally, novel approaches for CSP such as leadless LBBAP or bilateral bundle pacing are promising, but the safety, efficacy, and technology-specific advantages remain to be explored.

## Conclusion

CSP allows for a more physiological approach to CRT by recruitment of the native conduction system and studies in CSP demonstrate cumulative clinical evidence for its safety and efficacy in HF treatment. Although the clinical evidence from small RCT and observational studies is still insufficient to pose the CSP as a superior approach to BVP, the previous encouraging results underpin the prospect of the novel pacing modality as a primary CRT approach. The tailored candidates of CSP in CRT should be further defined by well-designed, prospective, randomized controlled studies with long-term follow-up and hard clinical outcomes including the mortality rate and HF hospitalization.
