# Supplementary material for: Recruitment of the cardiac conduction system for optimal resynchronization therapy in failing heart
Source: Front Physiol. 2022 Dec 15;13:1045740. doi: 10.3389/fphys.2022.1045740 (PMC9798297; doi:10.3389/fphys.2022.1045740)
Supplement: Supplementary file 1 [file Table1.pdf]

Supplemental Materials

Table S1: Summary of the clinical base in HBP for CRT

Study	Design	Study population	Sample size (N attempt for CSP)	Overall CSP success rates	Fluoroscopy Time (mean)	Pacing parameter (mean capture threshold and RWA)	Follow-up duration (months)	Electrical improvement (mean QRS duration: baseline to post-OP)	Functional improvement	Clinical improvement
Barba-Pichardo, 2013[1]	Prospective, single-center cohort	Symptomatic HF+LBBB	16	56%	23.4min	Not applicable	31.33	QRSd:166 to 97 ms	LVESD: 55.4 to 51.2 mm<br>LVEDD: 65.9 to 59.5mm<br>LVEF: 29% to 36%	NYHA class：<br>2 to 3
Daniel. Lustgarten, 2015[2]	Single-center randomization, cross-over study	QRSd>130ms HF with CRT indication	29	58.60%	na	1.2V @0.6ms	6	QRSd:169 to 131 ms (HBP)	26% to 32%	NYHA class:<br>2.9 to 1.9;<br>QOL: 54 to 38<br>6MWT: 269 to 383
OlujimiA. Ajijola, 2017[3]	Retrospective, multicenter cohort	BBB, QRSd>120ms, LVEF<35%, HF with CRT indication	21	76.2	na	1.9V@0.6ms	12	QRSd:181 to 129ms	LVEF：27% to 41%<br>LVEDD: 54mm-45mm	NYHA class：3.0 to 2.0
.Parikshit S. Sharma, 2018[4]	Retrospective, multicenter cohort	LVEF≤50% symptomatic HF	106	90	Na	1.4V @1.0ms;<br>RWA: 4mV	14	QRSd:163 to 113ms	LVEF: 30% to 44%	NYHA class：2.8 to 1.8
ParikshitS. Sharma,2018	Retrospective, multicenter cohort	LVEF≤50%,QRS d>120ms, RBBB	39	94.9	Na	1.1V @1.0ms<br>RWA:3.8 mV	15	QRSd:158-127ms	LVEF:31% to 39%	NYHAclass: 2.8 to 2

Peiren Shan, 2018[5]	Prospective, single-center cohort	pacing-dependent HF and LVEF< 50%	18	88.9	na	0.8V @0.5ms; RWA: 4.4 mV	12	QRSd:156.9 ms (RV PACING) to 107.1ms (HBP)	LVEF: 35.7% to 52.8%; LVEDD:62.3mm to 55.5mm	NYHA class: 3.1 to 1.3
Pugazhendhi Vijayaraman, 2019[6]	Retrospective, single-center cohort	PICM, RV lead failure, Lead system infection	85	93	10.8min	1.47V@1ms	25	QRSd： 177ms (RV pacing) to 115 ms (HBP)	LVEF: 34.3% to 48.2% in PICM patients (n=60)	NYHA class: 2.8 to 1.5
Weijian Huang , 2019[7]	Prospective, single-center cohort	LBBB, QRSd>130ms,symptomatic HF with CRT indication（n=74）	74	75.7	na	0.86V@0.5ms; RWA: 3.6mV	37.1	170.9 to 113.8ms	LVEF: 32.4% to 55.9% LVESV: 137.9 to 52.4mL	NYHA class:2.73to 1.03
Krzysztof Boczar, 2019[8]	Prospective, single-center cohort	AF, BBB , LVEF<40%, QRSd>130ms	14	97	na	na	14.4	159 to 128 ms	LVEF： 24% to 38% LVEDD:72 to 59mm LVESD： 59 to 47mm	NYHA class：3.07 to 1.65
Pugazhendhi Vijayaraman,2019[9]	Prospective, multicenter cohort	LBBB pattern , LVEF≤35%, NYHA 3-4	27	92.6	6.4min	1.7V @1.0ms	14	183ms to 151ms (HBP), 120ms (HOT-CRT)	HOT-CRT: LVEF：24% to 38% LVEDD: 65mm to 59mm	NYHA class: 3.3 to 2
GauravA. Upadhyay , 2019[10]	Multicenter RCT	QRSd>120ms , HF with CRT indication	26	61.5	na	2.25V@1.0ms	12	HBP: 174 to 125ms; BVP: 165 to 164 ms	HBP: LVEF: 28 to 34.6 %; BVP 27.7 to 32%	NYHA class improved >1: 53% in HBP vs 39 % in BVP, p=0.41; KCCQ HBP:+16, BVP +10, p=0.22

Pablo Morina-Va´zquez,2020[11]	Prospective, single-center cohort	LBBB , QRSd>130ms , HF with CRT indication	48	75	8.1min	1.6V@0.4ms	1	QRSd：160 to 132ms	LVEF: 30% to 51%	
Rajeev Singh , 2020	Retrospective, single-center cohort	LBBB-induced cardiomyopathy, LVEF< 40%	9	77.9	na	Na	14.5	QRSd:152 to115ms	LVEF：25% to 50%<br>LVESD：47mm to 37mm<br>LVEDD：55mm to 48mm	NYHA class：2.7-2
Amrish Deshmukh，2020[12]	Retrospective, single-center cohort	LBBB, QRSd>120ms or non-LBBB, QRSd>150ms, LVEF<35%	21	na	na	1.7V @0.8ms	32	QRSd: 170ms to 157ms (HBP) to 141ms (BVP) to 110ms (HOT).	LVEF: 27.6% to 41.1%；	NYHA class：3.1-2.1
Alwin Zweerink,2020[13]	Prospective, single-center cohort	BBB and HF with CRT indication	19	na	na	1.0V @0.4ms	Acute phase	QRSd：142 to 142ms (HBP), 154ms （optimized BVP), 126 ms (HOT-CRT)	-	
Michael Vinther，2021[14]	Single-center RCT	Strauss LBBB, LVEF≤35% , symptomatic HF with CRT indication	25	76	22min	1.8V @1.0ms	6	QRSd: 167 ms to 135ms (BVP);<br>163 to 129ms (HBP)	LVEF: 29% to 42% (BVP); 31 to 48% (HBP); p<0.05<br>LVESV: 131 ml to 83ml (BVP); 119 ml to 65 ml; p <0.05	NYHA class: 2.4 to 1.9 (BVP); 2.4 to 1.8 (HBP)

Jacopo Senes, 2021[15]	Retrospective, single-center cohort	HF with CRT indication	27	88.9	na	0.5V @0.5-1.0ms	9.6	QRSd: 163 to 128ms (HBP); 154ms to 148ms (BVP)	LVEF: 35.5% to 40.7% (HBP); 39.9 %-40.7% (BVP),	composite outcome rate: 11% in HBP group and 14% in BVP group.
Hiroyuki Kato, 2022[16]	Prospective, single-center cohort	LBBB,QRSd≥120ms, LVEF≤35%, HF with CRT indication	14	64.3	na	0.83@1.0ms; RWA: 2.8mV	12	QRSd：171.8 ms to 132.7ms (HBP); 180.4ms to 145.8ms (BVP)	LVEF: 21.2 % -51.7 % (HBP) 17.8%-29.2% (BVP) LVESV: 131.1ml to 41.2 ml （HBP）; 139.2ml to 95.6 ml（BVP）	NYHA class: 2.7-1.2 (HBP); 2.6 to 1.2 (BVP)

HF, heart failure; CSP, conduction system pacing; CRT, cardiac resynchronization therapy; HBP, His bundle pacing; BVP, biventricular pacing; RVP, right ventricular pacing; HOT-CRT, His-optimized CRT; OP, operation; RWA: R wave amplitude; LBBB, left bundle branch block; RBBB, right bundle branch block; QRSd, QRS duration; LVEF, left ventricular ejection fraction; LVEDD, left ventricular end-diastolic diameter; LVESD, left ventricular end-systolic diameter; LVESV, left ventricular end-systolic volume; PICM: pacing-induced cardiomyopathy; NYHA, New York Heart Association; AF, atrial fibrillation; QOL, Quality of life; 6MWT, 6-minute walk test; KCCQ, the Kansas City Cardiomyopathy Questionnaire.

Table S2: Summary of the clinical base in LBBAP for CRT

Study	Design	Study population	Sample size (number attempt for CSP)	Overall CSP success rates	Fluoroscopy Time (mean)	Pacing parameter (mean capture threshold and RWA)	Follow-up duration (months)	Electrical improvement (mean QRS duration: baseline to post-OP)	Functional improvement	Clinical improvement
Weiwei Zhang, 2019[17]	Prospective, single-center cohort	LBBB, LVEF≤40% HF with CRT indication	11	100	na	0.76V@0.4ms; RWA9.17 mv	6.7	QRSd: 180ms-129.09ms	100% have LVEF increased by over 5%, 63.6% have LVEF increased by over 20%, and LVESV by over 15%	81.8% have NYHA class reduced by 1
Jincun Guo, 2020[18]	Prospective, single-center cohort	Strauss LBBB, LVEF≤ 35%, symptomatic HF with CRT indication	24	87.5	na	0.48V@0.4ms	6	QRSd: 167.7 ms to111.7ms (LBBAP); 163.6-130.1 ms (BVP)	LVEF : 30%-50.9% (LBBAP); 29.8%-44.4% (BVP), p=0.15	NYHA class: 3.0to 1.3 (LBBAP); 3.0 to 1.5 (BVP)
Weijian Huang, 2020[19]	Prospective, multi-center cohort	LBBB, LVEF≤50%, HF with CRT indication	63	97	na	0.5V@0.5 ms; RWA 11.1mV	18	QRSd: 169 ms to 103ms	LVEF: 33% to 55%, LVESV: 123ml to 67ml	NYHA class:2.8 to 1.4

Xiaofei Li, 2020[20]	Prospective, multi-center cohort	LBBB, LVEF≤35%, HF with CRT indication	37	81.1	16.9min	0.81V@0.4ms	6	QRSd:178.2ms to to 121.8ms (LBBAP);180.9 ms to 158.7ms (BVP)	LVEF:28.8% to 44.3% (LBBAP) , 27.2% to 35%(BVP) LVEDD: 69.4 mm to 66.2mm (LBBAP) 66.5 to 59.2mm (BVP)	NYHA class 3.1 to 1.5 (LBBAP); 3.0 to 2.3 (BVP)
Yuqiu Li,2020[21]	Prospective, single-center cohort	HF with CRT indication	25	100	na	0.8V@0.5 ms; RWA 11.5mV	9.1	QRSd: 163.6-123ms	LVEF: 35.2% to 46.9% LVEDD : 64.1mm to56.8mm	NYHA class: 2.6 to 1.4
Floor C.W.M. Salden, 2020[22]	Prospective, multi-center cohort	HF with CRT indication	27	100	na	na	Acute phase	QRSd: 151ms to 135ms (LVSP) 136 ms (BVP), 134 ms (LBBAP+RV pacing), 110 ms (HBP)	LVdP/dtmax increased by 17% (LBBAP and BVP), by 11% (LBBAP +RV PACING), by 20% (HBP)	-
Pugazhendhi Vijayaraman, 2021[23]	Prospective, multi-center cohort	HF with CRT indication	325	85.2	na	0.8V@0.5 ms; RWA 10mV	6	QRSd: 152ms to 137ms	LVEF: 33% to 44%	NYHA class: 2.7 to 1.8

Yao Wang, 2020[24]	Prospective, single-center cohort	LBBB, LVEF≤35%，HF with CRT indication	10	100	na	0.54V@0.5ms	6	QRSd：183.6ms to 122.8ms (LBBAP); 174.6 ms to 141.6 ms (BVP)	LVEF：26.8 to 45.7% (LBBAP), 26.4% to 39.4% (BVP) LVEDD:68.6 mm to 57.5mm (LBBAP); 70.4mm to 61.6mm (BVP)	NYHA class: 2.9 to1.5 (LBBAP) 3.1 to 2.0 (BVP)
Xueying Chen，2021[25]	Prospective, multi-center cohort	LBBB, LVEF<40%, HF with CRT indication	50	98	9.5min	0.92V@0.5ms	12	QRSd：180.1ms-102.6ms; optimized BVP 175.7ms - 126.5ms	LVEF: 29.05 % to 49.1% (LBBAP), 28.36% to 43.6% (BVP) LVEDD: 60.07mm to 54.5 mm (LBBAP); 68.38 mm to 61 mm (BVP)	NYHA class III-IV，91.8% to 4.08% (LBBAP); 88.2% to 19.61% (BVP)
Wen Liu,2021[26]	Prospective, multi-center cohort	LBBB，LVEF≤35%, HF with CRT indication	34	79.4	na	na	4	QRSd：177.1ms to 113ms (LBBAP) QRSd narrowing by 64.1 in LBBAP by 32.1 in BVP, p<0.01	LVEF：29.9% to 47.1%，LVESD：56.6mm to 45mm，LVESV：141.4ml to 72.6ml； IVMD：56.4ms to28.9ms; PSD:143.4 ms to 92.6ms	NYHA class: 3.0 to 1.6, reduction in NYHA: -1.6 in LBBAP vs 0.9 in BVP

Shunmuga Sundaram Ponnusamy，2021[27]	Retrospective, multi-center cohort	LBBB-induced cardiomyopathy and heart failure	17	76.5	na	0.53V@0.5ms;RWA 11.7mV	12.70	QRSd:167.2ms to 110.4ms	LVEF:30.4% to 57.4%，LVEDD: 55.7 to 47.3mm	NYHA class:3.1 to 1.2
Lan Su, 2021[28]	Prospective, single-center cohort	Bradycardia and CRT indication	632, 88 LBBB	97.8	5.1min	0.65V@0.5ms; RWA:11mV	18.6	LBBB and HF: QRSd: 167.2ms to 124.0ms	LVEF: 48.82% to 58.12%	（QRSd>120ms）
Shengjie Wu，2021[29]	Prospective, single-center cohort	LBBB ，LVEF≤40% HF with CRT indication	96	82.3	5.2min	0.49@0.5ms; RWA:11.2mV	12	QRSd:170.3ms to 100.7ms (HBP); 168.8 ms to 104.3 ms (LBBAP), 161.1 to 135.8 ms (BVP)	LVEF increased by 23.9 % (HBP), 24% (LBBAP) and 16.7% (BVP)	NYHA class: 2.8 to 1.2 (HBP), 2.8 to 1.3 (LBBAP), 2.8 to 1.9 (BVP)
Linna Zu,2021[30]	Prospective, single-center cohort	LBBB (QRSd>150ms), LVEF < 35%，DCM	13	100	20.46min	0.9V@0.5ms; RWA:9mV	12	QRSd: 167.5 to 117.2 ms (LBBAP), 163.4 to 130.3 (BVP)	LVEF: 30.6% to 48.9% (LBBAP)，29.1% to 42.5% (BVP)	
Marek Jastrzebski, 2021[31]	Prospective, multi-center cohort	HF with CRT indication	112	81.3	27.3min	0.8V@0.5ms;RWA: 10mV	7.8	QRSd:182 ms to 144ms (LOT), 182ms to 170 ms (BVP); 182 ms to 162 ms (LBBAP)	LVEF: 28.5% to 37.2% (LOT); LVEDV: 209.8 ml -171.4 ml (LOT)	NYHA class: 2.9 to 1.9
Huacheng Li, 2021[32]	Prospective, single-center cohort	PICM	10	100	na	na	1	not specified	LVEF: 47.57% to 62.53%; LVEDD: 63.98mm to 55.24 mm	6MWT: 267.94 to 395.62ms

Zhiyong Qian, 2021[33]	Prospective, single-center cohort	symptomatic heart failure after pacemaker implantation	30	93.3	8.4min	0.6V@0.5 ms;RWA: 8.5mV	10.4	QRSd：174.1ms（RV pacing）to 116.6 ms (LBBAP)	LVEF:40.3% to 48.1%(PICM); 59.1% to 61.4% (HFpEF) LVESD: 46.8 mm to 41.6 (PICM); 34.8 mm to 33mm	NYHA class: 2.5 to 1.6
Yi-Heng, Yang, 2021[34]	Retrospective, single-center cohort	PICM	36	94	na	1.18v@0.4ms	11.52	QRSd : 184.22ms(RV pacing) to 120.52ms(CSP)	LVEF: 33.76% to 40.41%; LVEDD: 59.29 mm to 53.91mm	NYHA class: 2.55 to 2.00
Yang Ye, 2021[35]	Prospective, single-center cohort	infranodal AV block and PICM	20	95	7min	0.7V@0.4 ms;RWA: 12.7mV	12	QRSd：176.2ms（RV pacing）to 120.9 ms (LBBAP)	LVEF：36.3% to 51.9%; LVESV: 180.1ml to 136.8ml	NYHA class: 2.8 to 2.1
Leonard M[36] Rademakers	Prospective, single-center cohort	PICM	20	100	na	0.7V@0.4 ms;RWA: 9.1mV	6	QRS: 193-130ms	LVEF: 32% to 47%	NYHA class: 2.8 to 1.4
Leonard M Rademakers，2022[37]	Prospective, single-center cohort	NYHA II-IV, LVEF≤35%，Strauss LBBB	40	78	14min	0.8V@0.4 ms;RWA: 11.0mV	6	QRSd：166 to 123ms（LBBAP），	LVEF:28 to 43% (LBBAP) 31% to 41% (BVP)	

Marek Jastrzębski, 2022[38]	Multicenter registry	Bradycardia and CRT indication	696	82.2	9min	0.6V@0.5 ms;RWA: 10mV	6.4	na	241 CRT candidates: LVEF 31.5% to 39.4%; LVEDD: 60 mm to 57.4 mm	The complication rate of 8.3%
Pugazhendhi Vijayaraman 2022[39]	retrospective, multicenter, cohort	LVEF ≤35% + class II-IV heart failure symptoms, and met the criteria for CRT	258 CSP (HBP:87, LBBAP:171)	86%	16.8min	0.8V@0.5 ms;	27	QRSd: 150.5 to 133 ms	LVEF: 26.4% to 39.7% LVEDD：59 mm to 56.1 mm	Death or HFH is significantly lower with CSP vs BVP (28.3% vs 38.4%; hazard ratio 1.52; 95% confidence interval 1.082–2.087)
Pugazhendhi Vijayaraman 2022[40]	retrospective, multicenter, cohort	HF, RBBB, LVEF < 50%	121	88%	16min	0.8V@0.5 ms;RWA: 10mV	13	QRSd：156 ms to 150 ms	LVEF 35% to 43%	60% with clinical response
Pugazhendhi Vijayaraman 2022[41]	retrospective, multicenter, cohort	HF, BVP failure, or non-responder	212	94%	25.7min	0.69V@0.45ms;RWA: 10.4mV	12	QRSd: 170 ms to 139 ms	LVEF 29% to 40%	
Wang,Yao[42]	Prospective,	NICM,LBBB, LVEF ≤ 40%, , NYHA II-IV	40	90%	11.95min	0.69V@0.5 ms;	6	QRSd: LBBP:174.6ms to 131.5ms BVP:174.7ms to 136.6ms	LVEF: LBBP:28.7%to 52.0% BVP: 29.6% to 45.4%	NYHA reduction: -1.22

HF, heart failure; CSP, conduction system pacing; CRT, cardiac resynchronization therapy; LBBAP: Left bundle branch area pacing; HBP, His bundle pacing; BVP, biventricular pacing; RWA: R wave amplitude; RVP, right ventricular pacing; LOT-CRT, LBB-optimized CRT; OP, operation; BBB, bundle

branch block; LBBB, left bundle branch block; RBBB, right bundle branch block; AV, atrioventricular; QRSd, QRS duration; LVEF, left ventricular ejection fraction; LVEDD, left ventricular end-diastolic diameter; LVESD, left ventricular end-systolic diameter; LVESV, left ventricular end-systolic volume; NYHA, New York Heart Association; AF, atrial fibrillation; PICM: pacing-induced cardiomyopathy; QOL, Quality of life; 6MWT, 6-minute walk test; IVMD, interventricular mechanical delay; PSD, peak strain dispersion; HFpEF, heart failure with preserved ejection fraction; HFrEF, heart failure with reduced ejection fraction; HFH, heart failure hospitalization.

Table S3. Comparison of BVP HBP and LBBAP.

	**BVP**	**HBP**	**LBBAP**
**Technical efficiency**	Long fluoroscopy time.<br>Failure of the coronary sinus lead implantation.	Longer fluoroscopy time.<br>High technical challenge.	Shorter fluoroscopy time.<br>Shorter learning curve.<br>Higher successful rate.
**Pacing parameter**	Higher pacing threshold of LV lead.	Higher capture threshold.<br>Lower ventricular sensing.	Lower capture threshold.<br>Better ventricular sensing.
**Electrical synchrony**	Non-physiological activation than normal intrinsic conduction.	Similar physiologic activation of conduction system to intrinsic activation. Shorter QRS duration than BVP	Physiological LV activation.<br>Relatively delayed RV activation.<br>Shorter QRS duration than BVP.
**HF Population**			
Wide QRS complex			
LBBB	Adequate clinical evidence for validation.	Unable to correct block at the distal branch (Though distal HBP might overcome the limitation).<br>Similar clinical effect to BVP for Strauss LBBB from the small sample RCT.<br>More evidence from well-designed RCT is warrant.	Able to correct distal branch block.<br>Higher improvement in LVEF and QRSd reduction compared with BVP for Strauss LBBB and NICM. Long-term evidence from larger sample size RCTs is required.
RBBB	Less favorable clinical effect than LBBB.<br>But those with QRSd ≥ 150 ms might benefit.	Better RV activation than LBBAP and BVP from the computational study.<br>More clinical studies are needed.	Significant clinical and LVEF improvement from a retrospective study. Further prospective and comparative study are needed.
IVCD		Limited clinical study. Less favorable effect.	
Narrow QRS complex			

Expected high RV pacing proportion and cardiac dysfunction/ PICM	Recommended for upgrading therapy. Less favorable effect as primary therapy in HF and narrow QRSd.	Feasible and effective for upgrading therapy and a promising approach as primary therapy due to more physiological activation than BVP.	
AF and Ablation	Recommended by current guidelines.	Feasible, safe, and effective.<br>More significant LVEF improvement.	Feasible, safe, and effective, with similar clinical benefit. Fewer complications compared with HBP.

HF, heart failure; BVP, biventricular pacing; HBP, His bundle pacing; LBBAP: Left bundle branch area pacing; LV: left ventricular; RV, right ventricular; LBBB, left bundle branch block; RBBB, right bundle branch block; AV, atrioventricular; QRSd, QRS duration; LVEF, left ventricular ejection fraction; AF: atrial fibrillation; RCT: random controlled trial; NICM: non-ischemia cardiomyopathy; PICM: pacing-induced cardiomyopathy.

Reference

1. Barba-Pichardo R, Sanchez AM, Fernandez-Gomez JM, Morina-Vazquez P, Venegas-Gamero J, Herrera-Carranza M. Ventricular resynchronization therapy by direct His-bundle pacing using an internal cardioverter defibrillator. *EUROPACE*. 2013;15(1):83-88. doi:10.1093/europace/eus228

2. Lustgarten DL, Crespo EM, Arkhipova-Jenkins I, et al. His-bundle pacing versus biventricular pacing in cardiac resynchronization therapy patients: A crossover design comparison. *HEART RHYTHM*. 2015;12(7):1548-1557. doi:10.1016/j.hrthm.2015.03.048

3. Ajijola OA, Upadhyay GA, Macias C, Shivkumar K, Tung R. Permanent His-bundle pacing for cardiac resynchronization therapy: Initial feasibility study in lieu of left ventricular lead. *Heart Rhythm*. 2017;14(9):1353-1361. doi:10.1016/j.hrthm.2017.04.003

4. Sharma PS, Dandamudi G, Herweg B, et al. Permanent His-bundle pacing as an alternative to biventricular pacing for cardiac resynchronization therapy: A multicenter experience. *HEART RHYTHM*. 2018;15(3):413-420. doi:10.1016/j.hrthm.2017.10.014

5. Shan P, Su L, Zhou X, et al. Beneficial effects of upgrading to His bundle pacing in chronically paced patients with left ventricular ejection fraction < 50%. *HEART RHYTHM*. 2018;15(3):405-412. doi:10.1016/j.hrthm.2017.10.031

6. Vijayaraman P, Herweg B, Dandamudi G, et al. Outcomes of His-bundle pacing upgrade after long-term right ventricular pacing and/or

pacing-induced cardiomyopathy: Insights into disease progression. *Heart Rhythm*. 2019;16(10):1554-1561. doi:10.1016/j.hrthm.2019.03.026

7. Huang W, Su L, Wu S, et al. Long-term outcomes of His bundle pacing in patients with heart failure with left bundle branch block. *HEART*. 2019;105(2):137-143. doi:10.1136/heartjnl-2018-313415

8. Boczar K, Sławuta A, Ząbek A, et al. Cardiac resynchronization therapy with His bundle pacing. *Pacing Clin Electrophysiol*. 2019;42(3):374-380. doi:10.1111/pace.13611

9. Vijayaraman P, Herweg B, Ellenbogen KA, Gajek J. His-Optimized Cardiac Resynchronization Therapy to Maximize Electrical Resynchronization: A Feasibility Study. *Circ Arrhythm Electrophysiol*. 2019;12(2):e006934. doi:10.1161/CIRCEP.118.006934

10. Upadhyay GA, Vijayaraman P, Nayak HM, et al. His Corrective Pacing or Biventricular Pacing for Cardiac Resynchronization in Heart Failure. *J Am Coll Cardiol*. 2019;74(1):157-159. doi:10.1016/j.jacc.2019.04.026

11. Morina-Vazquez P, Teresa Moraleda-Salas M, Jose Manovel-Sanchez A, et al. Early improvement of left ventricular ejection fraction by cardiac resynchronization through His bundle pacing in patients with heart failure. *EUROPACE*. 2020;22(1):125-132. doi:10.1093/europace/euz296

12. Deshmukh A, Sattur S, Bechtol T, Heckman LIB, Prinzen FW, Deshmukh P. Sequential His bundle and left ventricular pacing for cardiac resynchronization. *J Cardiovasc Electrophysiol*. 2020;31(9):2448-2454. doi:10.1111/jce.14674

13. Zweerink A, Burri H. Next-level examination of His-optimized cardiac resynchronization therapy by noninvasive electrocardiographic activation mapping. *J Cardiovasc Electrophysiol*. 2020;31(11):3065-3066. doi:10.1111/jce.14736

14. Vinther M, Risum N, Svendsen JH, Møgelvang R, Philbert BT. A Randomized Trial of His Pacing Versus Biventricular Pacing in Symptomatic HF Patients With Left Bundle Branch Block (His-Alternative). *JACC Clin Electrophysiol*. 2021;7(11):1422-1432. doi:10.1016/j.jacep.2021.04.003

15. Senes J, Mascia G, Bottoni N, et al. Is His-optimized superior to conventional cardiac resynchronization therapy in improving heart failure? Results from a propensity-matched study. *Pacing Clin Electrophysiol PACE*. 2021;44(9):1532-1539. doi:10.1111/pace.14336

16. Kato H, Yanagisawa S, Sakurai T, et al. Efficacy of His Bundle Pacing on LV Relaxation and Clinical Improvement in HF and LBBB. *JACC Clin Electrophysiol*. 2022;8(1):59-69. doi:10.1016/j.jacep.2021.06.011

17. Zhang W, Huang J, Qi Y, et al. Cardiac resynchronization therapy by left bundle branch area pacing in patients with heart failure and left bundle branch block. *Heart Rhythm*. 2019;16(12):1783-1790. doi:10.1016/j.hrthm.2019.09.006

18. Guo J, Li L, Xiao G, et al. Remarkable response to cardiac resynchronization therapy via left bundle branch pacing in patients with true left bundle branch block. *Clin Cardiol*. 2020;43(12):1460-1468. doi:10.1002/clc.23462

19. Huang W, Wu S, Vijayaraman P, et al. Cardiac Resynchronization Therapy in Patients With Nonischemic Cardiomyopathy Using Left Bundle Branch Pacing. *JACC-Clin Electrophysiol*. 2020;6(7):849-858. doi:10.1016/j.jacep.2020.04.011

20. Li X, Qiu C, Xie R, et al. Left bundle branch area pacing delivery of cardiac resynchronization therapy and comparison with biventricular pacing. *ESC HEART Fail*. 2020;7(4):1711-1722. doi:10.1002/ehf2.12731

21. Li Y, Yan L, Dai Y, et al. Feasibility and efficacy of left bundle branch area pacing in patients indicated for cardiac resynchronization therapy. *EP Eur*. 2020;22(Supplement_2):ii54-ii60. doi:10.1093/europace/euaa271

22. Salden FCWM, Luermans JGLM, Westra SW, et al. Short-Term Hemodynamic and Electrophysiological Effects of Cardiac Resynchronization by Left Ventricular Septal Pacing. *J Am Coll Cardiol*. 2020;75(4):347-359. doi:10.1016/j.jacc.2019.11.040

23. Vijayaraman P, Ponnusamy S, Cano O, et al. Left Bundle Branch Area Pacing for Cardiac Resynchronization Therapy Results From the International LBBAP Collaborative Study Group. *JACC-Clin Electrophysiol*. 2021;7(2):135-147. doi:10.1016/j.jacep.2020.08.015

24. Wang Y, Gu K, Qian Z, et al. The efficacy of left bundle branch area pacing compared with biventricular pacing in patients with heart failure: A matched case-control study. *J Cardiovasc Electrophysiol*. 2020;31(8):2068-2077. doi:10.1111/jce.14628

25. Chen X, Ye Y, Wang Z, et al. Cardiac resynchronization therapy via left bundle branch pacing vs. optimized biventricular pacing with adaptive algorithm in heart failure with left bundle branch block: a prospective, multi-centre, observational study. *EP Eur*. Published online October 27, 2021:euab249. doi:10.1093/europace/euab249

26. Liu W, Hu C, Wang Y, et al. Mechanical Synchrony and Myocardial Work in Heart Failure Patients With Left Bundle Branch Area Pacing and Comparison With Biventricular Pacing. *Front Cardiovasc Med*. 2021;8. doi:10.3389/fcvm.2021.727611

27. Ponnusamy SS, Vijayaraman P. Left Bundle Branch Block–Induced Cardiomyopathy: Insights From Left Bundle Branch Pacing. *JACC Clin Electrophysiol*. 2021;7(9):1155-1165. doi:10.1016/j.jacep.2021.02.004

28. Su L, Wang S, Wu S, et al. Long-Term Safety and Feasibility of Left Bundle Branch Pacing in a Large Single-Center Study. *Circ Arrhythm Electrophysiol*. 2021;14(2). doi:10.1161/CIRCEP.120.009261

29. Wu S, Su L, Vijayaraman P, et al. Left Bundle Branch Pacing for Cardiac Resynchronization Therapy: Nonrandomized On-Treatment Comparison With His Bundle Pacing and Biventricular Pacing. *Can J Cardiol*. 2021;37(2):319-328. doi:10.1016/j.cjca.2020.04.037

30. Zu L, Wang Z, Hang F, et al. Cardiac resynchronization performed by LBBaP-CRT in patients with cardiac insufficiency and left bundle branch block. *Ann Noninvasive Electrocardiol*. 2021;26(6):e12898. doi:10.1111/anec.12898

31. Jastrzębski M, Moskal P, Huybrechts W, et al. Left bundle branch–optimized cardiac resynchronization therapy (LOT-CRT): Results from an international LBBAP collaborative study group. *Heart Rhythm*. 2021;0(0). doi:10.1016/j.hrthm.2021.07.057

32. Li H, Wang L, Peng X, Wu J. The quality of life of patients with pacemaker-induced cardiomyopathy after they upgrade to left bundle branch pacing. *Am J Transl Res*. 2021;13(4):3044-3053.

33. Qian Z, Wang Y, Hou X, et al. Efficacy of upgrading to left bundle branch pacing in patients with heart failure after right ventricular pacing. *Pacing Clin Electrophysiol*. 2021;44(3):472-480. doi:10.1111/pace.14147

34. Yang Y heng, Wang K xin, Ma P pei, et al. His-purkinje system pacing upgrade improve the heart performances in patients suffering from pacing-induced cardiomyopathy with or without permanent atrial fibrillation. *Int J Cardiol*. 2021;335:47-51. doi:10.1016/j.ijcard.2021.04.012

35. Ye Y, Wu S, Su L, et al. Feasibility and Outcomes of Upgrading to Left Bundle Branch Pacing in Patients With Pacing-Induced Cardiomyopathy and Infranodal Atrioventricular Block. *Front Cardiovasc Med*. 2021;8. doi:10.3389/fcvm.2021.674452

36. Rademakers LM, Bouwmeester S, Mast TP, Dekker L, Houthuizen P, Bracke FA. Feasibility, safety and outcomes of upgrading to left bundle branch pacing in patients with right ventricular pacing induced cardiomyopathy. *Pacing Clin Electrophysiol*. 2022;45(6):726-732. doi:10.1111/pace.14515

37. Rademakers LM, van den Broek JLPM, Bracke FA. Left bundle branch pacing as an alternative to biventricular pacing for cardiac resynchronisation therapy. *Neth Heart J*. Published online August 3, 2022. doi:10.1007/s12471-022-01712-9

38. Jastrzębski M, Kiełbasa G, Cano O, et al. Left bundle branch area pacing outcomes: the multicentre European MELOS study. *Eur Heart J*. Published online August 18, 2022:ehac445. doi:10.1093/eurheartj/ehac445

39. Fhrs PV. Clinical outcomes of conduction system pacing compared to biventricular pacing in patients requiring cardiac resynchronization therapy. *Heart Rhythm*. 2022;19(8):9.

40. Vijayaraman P, Cano O, Ponnusamy SS, et al. Left bundle branch area pacing in patients with heart failure and right bundle branch block: Results from International LBBAP Collaborative-Study Group. *Heart Rhythm O2*. Published online May 2022:S266650182200109X. doi:10.1016/j.hroo.2022.05.004

41. Vijayaraman P, Herweg B, Verma A, et al. Rescue left bundle branch area pacing in coronary venous lead failure or nonresponse to biventricular pacing: Results from International LBBAP Collaborative Study Group. *Heart Rhythm*. 2022;19(8):1272-1280. doi:10.1016/j.hrthm.2022.04.024

42. Wang Y, Zhu H, Hou X, et al. Randomized Trial of Left Bundle Branch vs Biventricular Pacing for Cardiac Resynchronization Therapy. *J Am Coll Cardiol*. 2022;80(13):1205-1216. doi:10.1016/j.jacc.2022.07.019
